# Experimental Virus Evolution Reveals a Role of Plant Microtubule Dynamics and TORTIFOLIA1/SPIRAL2 in RNA Trafficking

**DOI:** 10.1371/journal.pone.0105364

**Published:** 2014-08-18

**Authors:** Eduardo José Peña, Inmaculada Ferriol, Adrián Sambade, Henrik Buschmann, Annette Niehl, Santiago F. Elena, Luis Rubio, Manfred Heinlein

**Affiliations:** 1 Institut de Biologie Moléculaire des Plantes, UPR2357 CNRS, Strasbourg, France; 2 Instituto Valenciano de Investigaciones Agrarias, Moncada, Valencia, Spain; 3 Department of Comparative Neurobiology, Institut Cavanilles de Biodiversitat i Biologia Evolutiva CIBERNED, Universidad de Valencia, Valencia, Spain; 4 Institute for Botany, University of Osnabrück, Osnabrück, Germany; 5 Department of Environmental Sciences, Plant Physiology, University of Basel, Basel, Switzerland; 6 Instituto de Biología Molecular y Celular de Plantas, CSIC-UPV, Valencia, Spain; 7 The Santa Fe Institute, Santa Fe, New Mexico, United States of America; University of California, Riverside, United States of America

## Abstract

The cytoskeleton is a dynamic network composed of filamentous polymers and regulatory proteins that provide a flexible structural scaffold to the cell and plays a fundamental role in developmental processes. Mutations that alter the spatial orientation of the cortical microtubule (MT) array of plants are known to cause important changes in the pattern of cell wall synthesis and developmental phenotypes; however, the consequences of such alterations on other MT-network-associated functions in the cytoplasm are not known. *In vivo* observations suggested a role of cortical MTs in the formation and movement of *Tobacco mosaic virus* (TMV) RNA complexes along the endoplasmic reticulum (ER). Thus, to probe the significance of dynamic MT behavior in the coordination of MT-network-associated functions related to TMV infection and, thus, in the formation and transport of RNA complexes in the cytoplasm, we performed an evolution experiment with TMV in *Arabidopsis thaliana tor1/spr2* and *tor2* mutants with specific defects in MT dynamics and asked whether TMV is sensitive to these changes. We show that the altered cytoskeleton induced genetic changes in TMV that were correlated with efficient spread of infection in the mutant hosts. These observations demonstrate a role of dynamic MT rearrangements and of the MT-associated protein TORTIFOLIA1/SPIRAL2 in cellular functions related to virus spread and indicate that MT dynamics and MT-associated proteins represent constraints for virus evolution and adaptation. The results highlight the importance of the dynamic plasticity of the MT network in directing cytoplasmic functions in macromolecular assembly and trafficking and illustrate the value of experimental virus evolution for addressing the cellular functions of dynamic, long-range order systems in multicellular organisms.

## Introduction

Microtubules (MTs) are involved in a multitude of cellular processes such as intracellular transport and localization of organelles, determination of cell shape, or the perception and response to mechanical stimulus [1]. The plant MT network is highly dynamic and continuously remodeled into new arrangements in response to environmental and developmental information. In contrast to animal cells, where MTs are attached to the centrosome and extend with their polymerizing plus ends towards the cell periphery, cortical plant MTs are localized underneath the plasma membrane (PM) and form a barrel-shaped interphase array of dispersed MTs that do not share a common nucleation site. New MTs nucleate from mobile γ-tubulin-containing complexes that are, in most cases, recruited to existing MTs. The new MTs emerge either at a 40° angle or in parallel to the associated MTs and thus form either branched/crossover or interbundle arrangements, respectively. Upon nucleation, the new MTs may be severed away from their minus ends, thus creating free minus ends. The liberated minus ends of the severed MTs are now free to depolymerize which, if balanced by polymerization at the plus end, results in treadmilling and the translocation of the MTs along the PM [2]–[4]. MT severing also creates new plus ends that can regrow to elongated MTs at the crossover site [5]. Recent studies indicate that the severing activity of katanin at MT crossover sites is inhibited by the presence of TORTIFOLIA1/SPIRAL2 (TOR1), a MT-associated protein that promotes MT growth and stabilizes MT crossovers [6], [7]. By controlling MT severing, TOR1 appears to play a central role in regulating local MT patterning within the cortical array. Consistently, katanin and *tor1* mutants show alterations in the MT array. These and other mutations that affect the dynamic plasticity of the MT cytoskeleton also cause a wide range of developmental phenotypes thus illustrating the important role of MT array patterning during plant development [8]. However, although even slight changes in plant MT alignment caused by such mutations are known to affect growth, barely anything is known about the global consequences of such mutations on localized MT network-associated functions in the cytoplasm. It appears likely that the local patterning of MTs within the cortical array directs the local scaffolding for localized cellular functions and thus the functional and spatial organization of the cellular cortex. A role of MT patterning in directing localized functions of the cell is supported by specific local MT arrangements directing the patterns of cell wall synthesis in xylem and pavement cells [9]. Moreover, recent observations indicate that MTs are associated with endosomes and therefore could influence the abundance of membrane proteins such as PIN2 [10]. MTs linked to an endosomal pathway may also play a role in the targeting of non-cell-autonomous proteins to plasmodesmata (PD) [11]. MTs may also provide a framework for localized protein turnover processes such as ERAD (ER-associated degradation) [12], [13] and autophagy [14]. These and other observations suggest that locally organized MTs may facilitate the localized formation, maintenance, and turnover of PM domains and also of membrane-associated macromolecular complexes that are destined for transport to PD [15].

Consistent with the above-mentioned examples, cortical MTs are also implicated in the interaction of plants with cytoplasmic viruses and their targeting to PD. Among the plant viruses that have been reported to interact with MTs, *Tobacco mosaic virus* (TMV) is the best characterized [16]–[19]. TMV replicates its RNA genome in association with MT-associated sites of the cortical endoplasmic reticulum (ER) that together with the underlying actin network provides the structure along which the viral RNA is transported to PD and into adjacent cells. The virus moves its genome between cells in a non-encapsidated form and thus may rely on cellular mechanisms usually supporting the intra- and intercellular transport of endogenous RNA complexes. The virus-encoded movement protein (MP) that binds MTs [16], [20], [21] is essential for cell-to-cell movement of this virus [22]–[24], and the ability of the virus to spread between cells is correlated with the ability of MP to interact with MTs [17], [18]. Similar to RNA transport processes in other systems such as neurons or *Drosophila* oocytes [25]–[27], the transport of TMV RNA in plants is associated with the formation of mobile MP and RNA-containing particles/granules that are transported in a stop-and-go manner [18], [28]. *In vivo* studies with conditional mutations in MP demonstrated that the formation of the granules is functionally linked to the ability of the protein to bind MTs and to its function in viral RNA movement [18], [28]. Consistent with a role of MT binding, the granules move along the cortical ER and pause their movements at MT-associated ER sites [28]. The granules observed in cells at the infection front of the virus are proposed to represent early viral replication complexes (VRCs) and either spread along the ER-actin network to reach PD or remain in the infected cell to give rise to large VRC clusters or “virus factories” that are observed during later infection stages [19]. The virus factories contain replicase, viral RNA, as well as MP, and accumulate the viral coat protein (CP) and virions on the surface [29]. They are associated with the ER-actin network as well as with numerous branching MTs suggesting that the assembly and growth of VRCs may involve local rearrangements in the cortical MT array [19]. The local MT rearrangements may reflect a function of MP since this protein appears to be capable to hijack the MT nucleation machinery [17], [19], [28], [30], [31]. By inducing such local MT rearrangements, the MP may support the formation, trafficking, and further maturation of VRCs by creating a local scaffold for VRC anchorage and the recruitment of host factors and ER membranes [15], [19].

However, although the *in vivo* observations suggest a role of dynamic plasticity in the MT cytoskeleton for virus replication and movement, additional tools are required to support this model. Here, we decided to make use of the high mutation rate and evolvability of RNA viruses [32] to determine the importance of MT dynamics and patterning in the cortical MT array for virus movement by experimental virus evolution. Indeed, if the above model is valid, the dynamic plasticity of the MT cytoskeleton should be vital for the virus and any important change in the dynamic plasticity of the MT cytoskeleton should produce a selective pressure for the virus to evolve. Such evolutionary response would demonstrate the role of MT rearrangements during infection and would highlight the value of TMV evolvability as a system for testing the role of the MT cytoskeleton and its dynamic plasticity in directing cytoplasmic functions, particularly in the formation and trafficking of RNA complexes.

To test the functional intimacy between TMV and the MT cytoskeleton and particularly the role of the dynamic MT cytoskeleton in directing cytoplasmic functions related to the development and transport of viral RNA complexes, we challenged TMV with *tortifolia 1/spiral 2* (*tor1*) and *tortifolia 2* (*tor2*) mutants with specific defects in the dynamic behavior of MTs within the cortical MT array. Whereas the *tor1* mutant is a knock-out of the MT-associated protein TOR1 and is inhibited in efficient MT polymerization and in the formation and maintenance of MT crossovers [6], *tor2* carries a conservative arginine to lysine change at position 2 of the primary amino acid sequence (R2K mutation) of the α-tubulin 4 protein that is proposed to interfere with contacts between R2 of α-tubulin and the GTPase domain of β-tubulin and thus to reduce the rate of MT polymerization [33]. In this work, we demonstrate that the virus is rather well adapted to the *A. thaliana* wild type. However, the virus is highly sensitive to *tor1* and *tor2* mutations and responds to the respective changes in the dynamic plasticity of the MT cytoskeleton with mutations that are correlated with efficient spread within the mutant hosts without significantly increasing the efficiency of virus replication. The results demonstrate the importance of dynamic MT network plasticity for the establishment of cytoplasmic functions required for efficient TMV movement and that MT dynamics and MT-associated proteins represent constraints for virus evolution and adaptation. The results suggest an important role of TOR1, which may stabilize MT crossovers to support the formation and intercellular spread of the viral RNA complexes. We propose that these findings reflect the importance of the dynamic MT cytoskeleton in the formation and transport of viral as well as endogenous RNA complexes.

## Results

**Figure 1:**
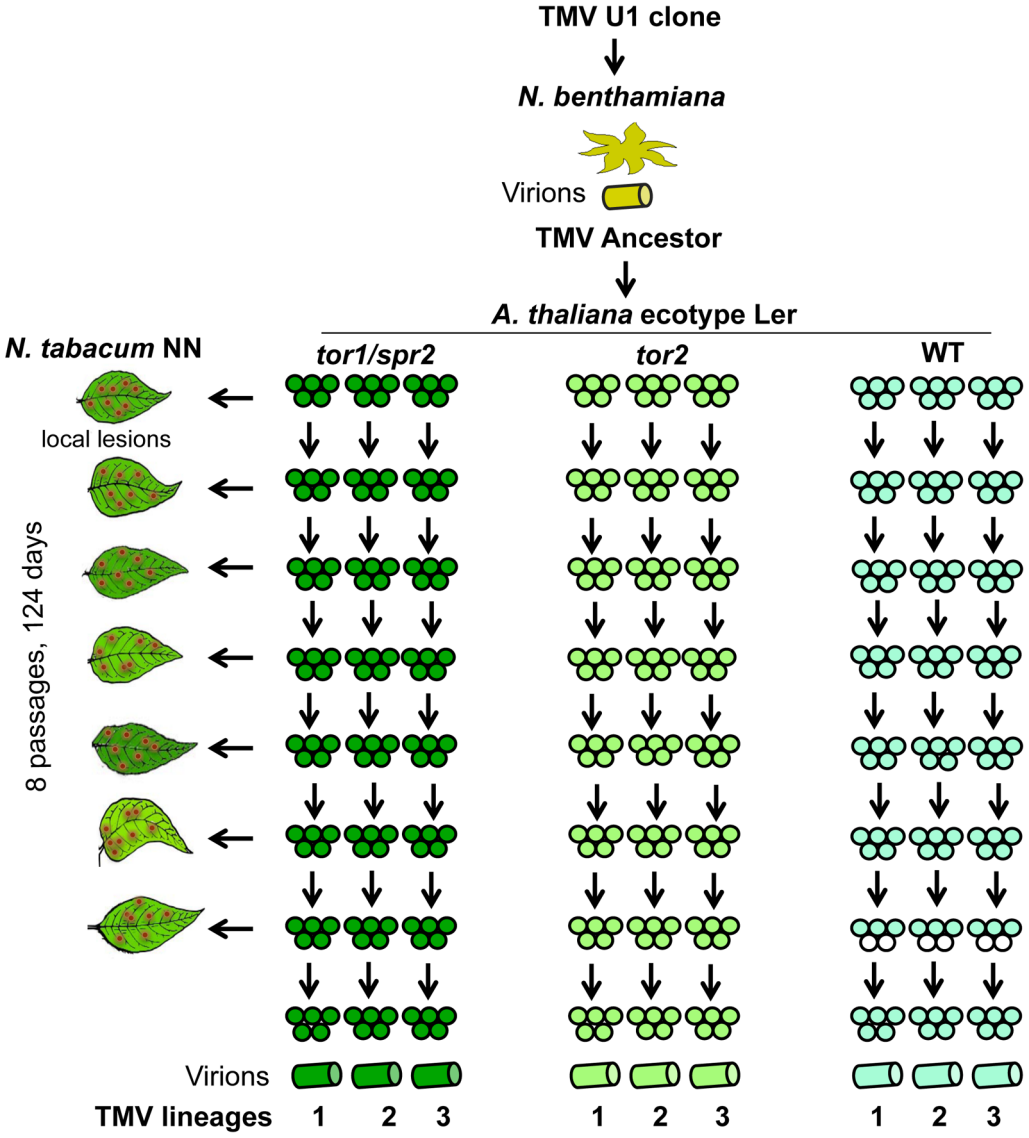
Layout of the virus evolution experiment. The initial TMV inoculum (ancestor) was obtained from *N. benthamiana* plants inoculated with a cDNA clone of TMV and was used for inoculation of *tor1/spr2, tor2,* and wild type (WT) *A. thaliana* plants (three sets of five plants each). Following eight passages, nine independent TMV lineages were obtained. The number of infectious particles used for each passage was controlled by local lesion assays with hypersensitive *N. tabacum* NN plants.

To test the response of TMV to alterations in MT network dynamics, we used a TMV isolate obtained after replication of a TMV cDNA clone [24] in *Nicotiana benthamiana* plants (Figure 1). Using this isolate as the ancestral virus in our evolution experiment, we inoculated three sets (replicates) of five wild type, five *tor1,* and five *tor2 A. thaliana* plants. At 15 days post inoculation (dpi), the non-inoculated, systemically infected, young leaves of all plants of a given set were pooled and used for virus extraction. The respective virus extracts were then used to inoculate new sets of plants. This passaging from one plant generation to the next was performed eight times. The number of infectious virion particles in each inoculum was estimated by quantifying the number of local cell death lesions following inoculation of hypersensitive *N. tabacum* NN plants (Figure S1). These tests demonstrated that the inoculum carried 150 to 400 infectious virion particles in all passages thus indicating the absence of strong bottlenecks during passages that could favor the accumulation of mutations by genetic drift and hinder fixation of adaptive mutations. After the eighth passage, nine final viral lineages were created (Figure 1): three lineages from *tor1* plants (Tor1-1, Tor1-2 and Tor1-3), three lineages from *tor2* plants (Tor2-1, Tor2-2 and Tor2-3), and three lineages from wild type plants (WT-1, WT-2 and WT-3).

**Figure 2:**
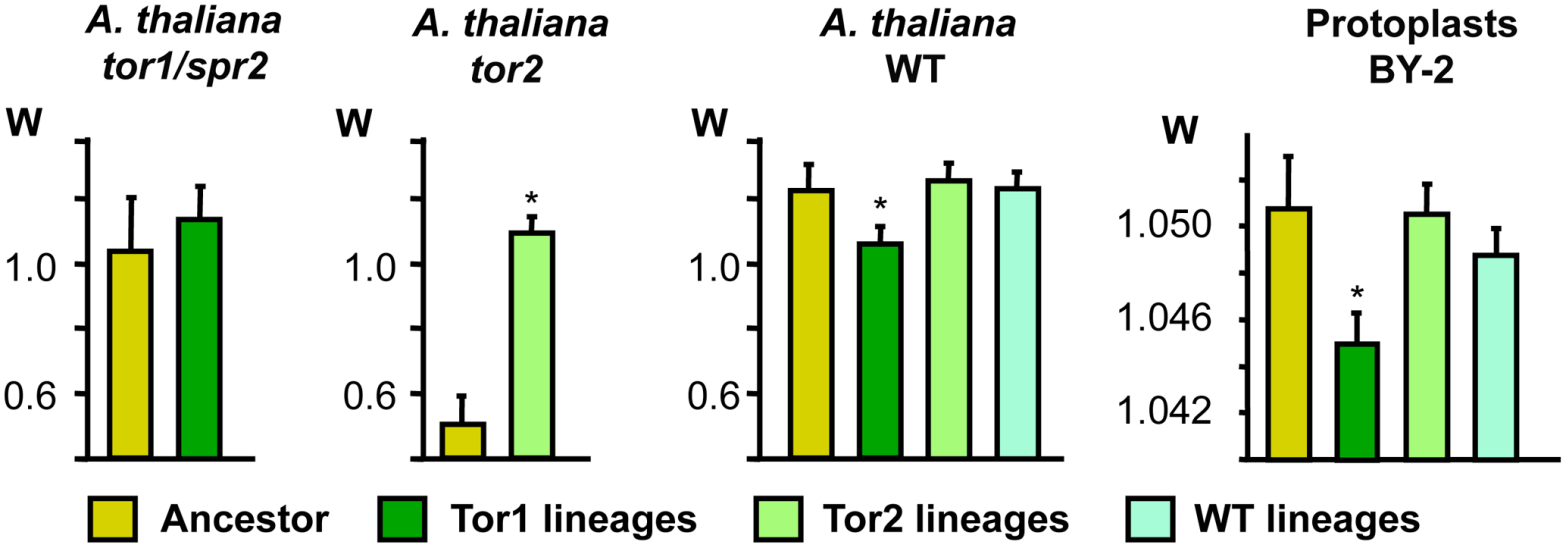
Average absolute fitness (*W*) of the TMV ancestor and evolved lineages. *W* is shown for infection in *A. thaliana tor1/spr2, tor2* and WT plants, and in BY-2 protoplasts. Tor2 lineages show adaptation for efficient systemic movement in *tor2* plants without consequences on replication efficiency (lack of a change in *W* compared to ancestor in protoplasts). Tor1 lineages are affected in replication efficiency and show slight adaptation for efficient systemic movement in *tor1/spr2* plants. Asterisks indicate statistically significant differences from the ancestor.

To evaluate adaptation of TMV to each host genotype, the fitness of each experimentally evolved viral lineage was compared with that of the ancestral virus. Absolute fitness, *W,* was determined by measuring the over-time accumulation of viral RNA in the upper, non-inoculated leaves as described in the Materials and Methods section. Whereas the level of viral accumulation in plants, and thus *W*, results from intracellular replication and the number of new cells infected by intercellular movement, viral accumulation in protoplasts is determined only by intracellular replication. Thus, to distinguish the contribution of intercellular RNA movement from that of RNA replication, viral accumulation was measured in three sets (replicates) of the wild type and mutant plants as well as in protoplasts upon inoculation with the respective viral lineage or the ancestral virus. The results indicate that relative to the ancestral virus the *tor1*-derived lineages developed a significant decrease in *W* in wild type plants (13%; P = 0.035) and a slight, yet statistically significant reduction of *W* in BY-2 protoplasts (0.53%; P = 0.031). Nevertheless, the same *tor1*-derived lineages showed a trend of higher *W* in *tor1/spr2* plants (8.4%) (Figure 2). These results indicate that the mutant cytoskeleton in *tor1/spr2* plants triggered changes in TMV that allowed TMV to adapt and thus to maintain or even increase fitness, i.e. viral RNA movement efficiency, in this environment. However, as shown by the decrease of *W* in BY-2 protoplasts, the adaptive changes induced by the mutant cytoskeleton in *tor1* plants caused a fitness trade-off for replication. The reduced RNA replication efficiency of the *tor1*-derived lineages likely contributed to the decrease of *W* in wild type plants and may have prevented a more significant increase of *W* in *tor1* plants. The ability of *tor1* to trigger adaptation in TMV indicates a strong impact of dynamic MT behaviour and of TOR1 on virus replication and movement.

The *tor2*-derived lineages showed a statistically significant average increase in *W* of 72.2% in *tor2* plants (*P*<0.001) whereas *W* remained similar to that of the ancestral virus in the wild type plants and protoplasts (Figure 2). Thus, unlike *tor1*, the presence of *tor2* induced TMV adaptation without causing a trade-off in viral replication efficiency. The adaptation of TMV to *tor2* supports a role of MT polymerization efficiency in TMV movement. Finally, the fitness of WT-derived lineages remained similar to that of the ancestral virus in wild type plants and protoplast (Figure 2 and Table S1) indicating that the common strain of TMV (U1) is well adapted to *A. thaliana.*

Sequence analysis for each of the nine lineages revealed 11 nucleotide substitutions in seven lineages with respect to the ancestral virus (Table 1). The *tor1/spr2*- and *tor2*- derived lineages showed one or two nucleotide substitutions in the first open reading frame (ORF) of the viral RNA, which encodes the 126 kD and 183 kD subunits of the replicase (Table 1). Nucleotide substitutions in lineages Tor1-1, Tor1-3 and Tor2-3 caused amino acids changes whereas the Tor2-1 and Tor2-2 lineages had synonymous substitutions suggesting that in addition to changes in the protein also the secondary structure of the viral RNA or its translational efficiency may play a role in optimizing viral fitness.

**Table 1:** Nucleotide substitutions in evolved TMV lineages.

	Rep^b^					MP^b^	CP^b^
Lineages^a^	MT^c^	HEL^c^			POL^c^		
**Tor1-1**	-	-	A2542G, T3235C	-	-	-	-
**Tor1-2**	-	-	-	-	-	-	-
**Tor1-3**	-	-	-	C3344T	-	-	-
**Tor2-1**	-	-	T2627C	-	T4293C*	-	-
**Tor2-2**	-	T1827C*	-	-	-	-	-
**Tor2-3**	C693A^d^	-	-	-	-	-	-
**WT-1**	-	-	-	-	-	-	-
**WT-2**		-	T2627C	-	-	-	-
**WT-3**	A764G, T798C*	-	-	-	-	-	T180C*

a TMV lineages obtained after eight serial passages of the TMV ancestor through *A. thaliana tor1/spr2*, *tor2,* and wild type (WT).

b ORF-encoded proteins; Rep, 126 k/183 k replicase; MP, movement protein; CP, coat protein.

c Domains of the 126 k/183 k replicase; MT, methyltransferase domain; HEL, helicase domain; POL, RNA-dependent RNA polymerase domain. The shorter 126 k subunit lacks the POL domain.

d Nucleotide positions are relative to the first nucleotide of each ORF. Synonymous substitutions are indicated by asterisks.

## Discussion

The cortical interphase MT array of plants is well known to drive the insertion [34], [35] and trajectory of the cellulose synthase complex at the plasma membrane [36], [37] and thereby direct the parallel deposition of cellulose microfibrills that mediates anisotropic growth [38], [39]. However, there is only very limited information about the role of the dynamic MT array in guiding other MT-associated functions in the cytoplasm. Our previous studies established that MTs play a role during TMV infection and undergo interactions with the virus-encoded MP, and that these interactions are important for the cell-to-cell movement of the virus [17], [18], [21]. *In vivo* observations indicated that MTs are associated with VRCs functioning as replication factories during late infection as well as with early VRCs, thus with MP-associated RNA complexes functionally associated with the cell-to-cell movement of the virus [19], [28]. To gain further insight into the role of the MT cytoskeleton and particularly of its dynamic plasticity in guiding cytoplasmic functions related to TMV infection, we challenged TMV with specific *A. thaliana* mutants affected in MT dynamics. Whereas *tor1/spr2* interferes with MT orientation and growth [7], [40] and the stabilization of MT crossovers [6], *tor2* affects tubulin polymerization efficiency [33]. We found that TMV is sensitive to these mutations and undergoes adaptive evolution that allows it to enhance its fitness in the respective mutant hosts. The ability of the host mutations to induce adaptive changes in the virus demonstrates the importance of the dynamic plasticity of the MT cytoskeleton for guiding MT network-related functions in the cytoplasm, i.e. functions that are required to support virus infection.

Given that the mutant host environment induced viral adaption and increases in fitness with respect to systemic spread without involving increases in viral replication efficiency in any of the evolved lineages, the observations indicate a role of dynamic MT network functions in TMV RNA movement. Viral adaptation to *tor1* and to *tor2* correlated with the fixation of mutations clustering in the replicase-encoding ORF of the virus, thus indicating a role of the replicase subunits in these processes. Although previous evidence supports a role of the replicase in TMV spread [41], an interaction of these proteins with MTs has not been reported. The observation that host mutations affecting the dynamic rearrangements within the MT network caused changes in the replicase is consistent with the role of the 126 k replicase protein in regulating the size of the VRCs [42] and with current models implying a role of MTs in the anchorage and assembly of these complexes [19]. Interestingly, no adaptive mutations were found in the MP, which interacts with MT *in vivo* and *in vitro* [21], or in the coat protein, which is required for TMV long distance movement through the phloem [43]. This may suggest that the *tor1* and *tor2* mutations do not interfere with the interactions of MP with tubulin but rather impair the important role of local MT patterning and scaffolding in the formation, movement and further maturation of VRCs. The CP was reported to control VRC growth by regulating the production of subgenomic RNAs and thus the amount of MP that accumulates in VRCs. However, this protein does not interact with MTs, is dispensable for virus replication, and accumulates outside the VRCs [29] and may therefore not be involved in MT-related mechanisms that interact with the VRCs for movement.

Viral adaptation in response to *tor1* and *tor2* reveals the importance of local MT patterning and associated functions in the formation and transport of viral and potentially endogenous RNA complexes. Since the mutations in *tor1/spr2* and *tor2* plants interfere with MT dynamics through different mechanisms, these processes do not necessarily depend on direct interactions with tubulin and SPIRAL2 but rather on their function in controlling the local MT network superstructure. Whereas the viral adaptation to both host mutations reflects the importance of normal MT dynamics, the adaptation to *tor1* indicates the specific importance of TOR1 function. The function of TOR1 in maintaining MT crossovers during infection may be consistent with evidence indicating that MP hijacks MT nucleating complexes [30]. According to current hypothesis, the MP recruits MT nucleating complexes to early VRCs to generate a MT scaffold required for VRC maturation and growth, thus leading to the MT crossovers associated with VRCs seen *in vivo* [19]. TOR1 function may be required to suppress MT severing by katanin [6], which would otherwise act against the MT arrangement induced by MP. That a reduced MT polymerization efficiency and a reduced capacity of the cell to maintain MT crossovers lead to changes in the replicase suggests that these changes in MT activities affect VRC structure, movement, or maturation and that the deficiencies imposed by the mutations can be complemented by the structural composition of the VRC, which may be determined by the structure of the replicase or the viral RNA. The range of the genetic changes in the adapted virus lineages suggests that the respective replicase and RNA domains offer several options and plasticity for adaptation to the alteration in MT dynamics. This plasticity in the viral genome may play an important role in allowing the virus to replicate and move between different cell types and hosts.

Our results demonstrate that the U1 strain of TMV is well adapted to the model plant *A. thaliana* and that the interaction of TMV with the altered hosts exerted a positive selection of gain-of-function mutations in the viral genome which allowed efficient viral RNA spread. The capacity of TMV to undergo adaptive evolution (evolvability) in response to altered host components commends its suitability for experimental viral evolution experiments to identify and determine the significance of specific gene functions in relation to dynamic membrane and cytoskeleton-related processes that are involved in infection and that may as well also play fundamental roles during plant development and disease.

## Materials and Methods

### Virus production

To generate the initial inoculum for the virus evolution experiment, a full-length cDNA clone of TMV strain U1 [24] was linearized with Acc65I and capped infectious TMV RNA was *in vitro* transcribed using the RiboMax Large Scale RNA production system-T7 (Promega). Three *N. benthamiana* plants (3 to 4 weeks old) were infected by mechanical inoculation of the second true leaf with 4 µg viral RNA per plant, in the presence of Celite 545 (Prolabo). Following inoculation, the plants were maintained in a greenhouse at 16 h light/8 h dark cycles at 22/18°C. Virion particles were purified and quantified from symptomatic tissue at 7 dpi using published methods [12].

### Arabidopsis plants and inoculation

Wild type, *tor1* [40] and *tor2* [33] *A. thaliana* (ecotype Landsberg *erecta*) plants were germinated and cultivated in growing chambers with 16 h light/8 h dark cycles at 22/18°C. The 5^th^ and 6^th^ leaves of 4 weeks old plants were rub-inoculated with virions in the presence of Celite 545 (Prolabo). Inoculated plants were maintained under greenhouse conditions, with 16 h light/8 h dark cycles at 22/18°C.

### Viral passages in Arabidopsis

The virus evolution experiment involved eight viral passages between generations of plants, each consisting of three sets (replicates) of five plants for each plant genotype (Figure 1). For each passage at 15 dpi, three upper leaves of the systemically infected plants in each set were harvested and pooled. Viruses were extracted from these pools by addition of 3 ml of 10 mM sodium phosphate buffer (pH 7.0) per 1 g of infected plant tissue, followed by homogenization using a Precellys tissue lyser (Bertin). The extracts were clarified by centrifugation for 10 min at 12000×g and used for inoculation of new plants. The titer of infectious viral particles in the inoculum of each passage was determined by counting the number of local cell death lesions following inoculation of hypersensitive *N. tabacum* NN plants.

### Analysis of viral fitness

The fitness of each viral lineage and the ancestral virus was analyzed by inoculation of *A. thaliana* wild type, *tor1/spr2* and *tor2* plants (10–13 plants each). Plants were inoculated with 1 µg of purified virions and non-inoculated upper leaves were harvested at 7, 12 and 15 dpi to evaluate systemic infection. Harvested samples were used for TMV detection by a commercial DAS-ELISA kit (DSMZ RT-0041, Germany, using the manufacturer's instructions) and for analysis of viral RNA titer in ELISA-positive plants. RNA titer was determined by quantitative RT-PCR [44] using the TaqMan probe 6-FAM-AGACAGCCCACATGTTTTGGTCGCA and primers TMV-3093F **(** CCGATCTCAAAACCCTTGCA) and TMV1R (CGAACAGGTGTGCCTTGACA). Viral RNA titers (Table S1) were used to calculate absolute fitness, *W*, defined as *W* = *e^r^*, where *r*, the Malthusian growth rate, was estimated by regression of the logarithm of the number of viral RNA molecules that accumulate over time (7, 12 and 15 dpi). *W* was analyzed by using a Generalized Linear Model [45]. All *W* calculations refer to times after infection during which the virus was actively spreading into non-inoculated leaves.

To determine replication efficiency in the absence of virus movement, *W* was measured in infected tobacco Bright Yellow-2 (BY-2) protoplasts [46]. Protoplasts (1×10^6^ cells) were inoculated with 1 µg of purified virions using electroporation [47]. Samples were collected at 0, 6, 12, 24, 48 and 72 hours post inoculation and used for total RNA extraction and subsequent analysis by quantitative RT-PCR. Three independent experiments (biological replicates) were performed for each viral lineage and the ancestor. The results of these quantifications are shown in Table S2.

### Nucleotide sequencing

The consensus nucleotide sequences of the TMV ancestor and the nine TMV lineages were obtained by sequencing of reverse-transcribed and PCR-amplified viral RNA extracted from virions (purified as above) belonging to the 8th passage. Viral RNA was purified from virions by a protocol involving phenol/chloroform/isoamyl alcohol extraction followed by isopropanol precipitation [48]. Nine pairs of primers mapping along the TMV genome were used (Table S3). Reverse transcription was performed at 42°C for 1 h with SuperScriptIII Reverse Transcriptase (Invitrogen), followed by PCR amplification using iProof High-Fidelity DNA Polymerase (BIO-RAD) in a GeneAmp PCR System 9700 (Applied Biosystems) and applying 2 min at 94°C, 35 cycles of 15 s at 94°C, 15 s at 55°C and 40 s at 72°C, and a final incubation for 5 min at 72°C. PCR products were purified using Illustra GFX PCR DNA and Gel Band Purification kit (GE Healthcare). Nucleotide sequences were determined with a 3130xL Genetic Analyzer (Applied Biosystems) and assembled with the program STADEN 2.0.0b9 [49]. Nucleotide sequences were deposited at GenBank under accession numbers KF972427-KF972436.

## Accession numbers

Nucleotide sequences were deposited at GenBank under accession numbers KF972427-KF972436.

## Supporting Information

Figure S1 **Local necrotic lesion assay using hypersensitive tobacco NN plants to estimate the number of infectious particles in a plant extract.** The left half of the leaf was inoculated with a control extract from a healthy *A. thaliana* plant. The right half of the leaf was inoculated with extracts from a TMV-infected *A. thaliana* plant (WT-1 from the fourth passage). The number of lesions reflects the number of particles in the inoculum.
(JPG)

Table S1 **Accumulation of the TMV ancestor and evolved lineages in non-inoculated, systemic leaves in** ***tor1/spr2*** **,** ***tor2*** **, and WT plants at 7, 12 and 15 dpi.**
(DOCX)

Table S2 **Accumulation of the TMV ancestor and evolved lineages in tobacco BY-2 protoplasts at 6, 12, 24, 48 and 72 hpi.**
(DOCX)

Table S3 **Forward (F) and reverse (R) primers used for TMV genome amplification by RT-PCR and nucleotide sequencing.**
(DOCX)
