# Supplementary figures and images for: Experimental Virus Evolution Reveals a Role of Plant Microtubule Dynamics and TORTIFOLIA1/SPIRAL2 in RNA Trafficking
Source: PLoS One. 2014 Aug 18;9(8):e105364. doi: 10.1371/journal.pone.0105364 (PMC4136834; doi:10.1371/journal.pone.0105364)

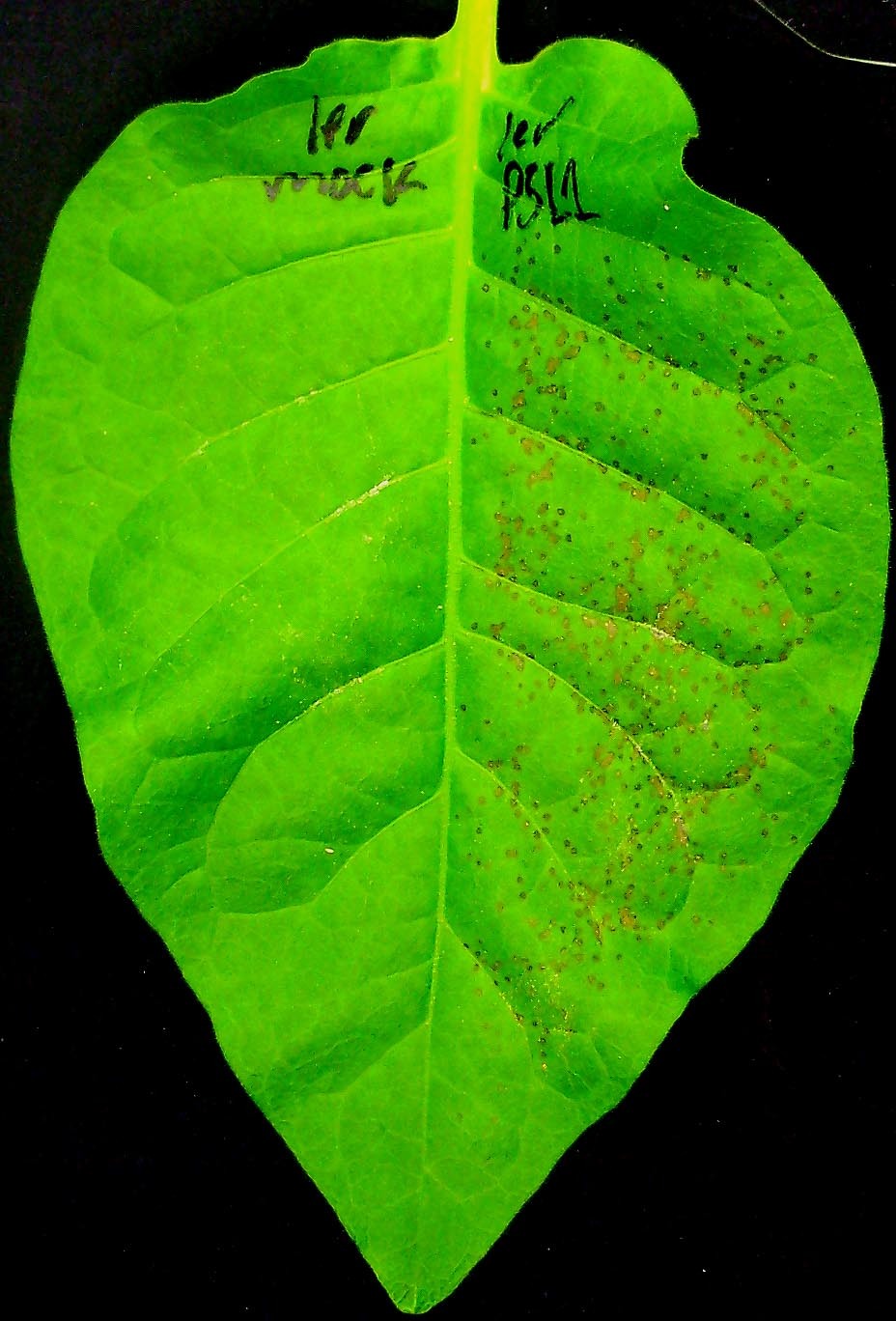

Supplement: Figure S1 — Local necrotic lesion assay using hypersensitive tobacco NN plants to estimate the number of infectious particles in a plant extract. The left half of the leaf was inoculated with a control extract from a healthy A. thaliana plant. The right half of the leaf was inoculated with extracts from a TMV-infected A. thaliana plant (WT-1 from the fourth passage). The number of lesions reflects the number of particles in the inoculum. (JPG) [file pone.0105364.s001.jpg]
