# Supplementary material for: Experimental Virus Evolution Reveals a Role of Plant Microtubule Dynamics and TORTIFOLIA1/SPIRAL2 in RNA Trafficking
Source: PLoS One. 2014 Aug 18;9(8):e105364. doi: 10.1371/journal.pone.0105364 (PMC4136834; doi:10.1371/journal.pone.0105364)
Supplement: Table S1 — Accumulation of the TMV ancestor and evolved lineages in non-inoculated, systemic leaves in tor1/spr2 , tor2 , and WT plants at 7, 12 and 15 dpi. (DOCX) [file pone.0105364.s002.docx]

**Table S1.** Accumulation of the TMV ancestor and evolved lineages in non-inoculated, systemic leaves in *tor1/spr2*, *tor2*, and WT plants at 7, 12 and 15 dpi

|  |  |  | **Number of viral RNA copies (mean ± SE)** | | |
| --- | --- | --- | --- | --- | --- |
| Host | Virus | **R** | **7** | **12** | **15** |
| ***tor1/spr2*** | **Ancestor** | 3 | 1.1±0.5 ×10^8^ | 2.7±1.3 ×10^7^ | 4.0±3.0 ×10^7^ |
|  | **Tor1-1** | 3 | 6.2±3.1 ×10^6^ | 1.3±0.5 ×10^7^ | 4.1±2.1 ×10^7^ |
|  | **Tor1-2** | 3 | 2.7±2.6 ×10^5^ | 2.8±0.6 ×10^7^ | 6.0±1.6 ×10^7^ |
|  | **Tor1-3** | 3 | 3.1±3.0 ×10^7^ | 4.1±0.8 ×10^7^ | 2.4±0.4 ×10^6^ |
| ***tor2*** | **Ancestor** | 3 | 1.5±2.1 ×10^7^ | 2.5±2.8 ×10^6^ | 1.0±2.1 ×10^7^ |
|  | **Tor2-1** | 3 | 8.8±3.5 ×10^6^ | 1.3±0.4 ×10^8^ | 5.6±0.2 ×10^6^ |
|  | **Tor2-2** | 3 | 8.5±7.2 ×10^6^ | 1.2±0.5 ×10^8^ | 6.3±5.5 ×10^7^ |
|  | Tor2-3 | 3 | 4.5±2.1 ×10^5^ | 9.0±4.4 ×10^7^ | 2.1±0.9 ×10^7^ |
| **WT** | Ancestor | 3 | 5.8±5.3 ×10^4^ | 4.2±0.7 ×10^6^ | 4.2±2.2 ×10^6^ |
|  | **Tor1-1** | 3 | 2.3±1.0 ×10^5^ | 4.5±1.6 ×10^7^ | 1.7±0.7 ×10^7^ |
|  | **Tor1-2** | 3 | 2.1±1.1 ×10^7^ | 6.9±5.0 ×10^6^ | 1.5±0.6 ×10^7^ |
|  | **Tor1-3** | 3 | 2.9±1.9 ×10^5^ | 1.8±0.8 ×10^7^ | 1.5±0.1 ×10^7^ |
|  | **Tor2-1** | 3 | 9.9±3.3 ×10^4^ | 3.1±1.2 ×10^7^ | 1.6±0.8 ×10^8^ |
|  | **Tor2-2** | 3 | 6.8±4.0 ×10^4^ | 4.5±2.9 ×10^7^ | 1.4±0.6 ×10^8^ |
|  | **Tor2-3** | 3 | 3.0±0.9 ×10^5^ | 2.8±1.3 ×10^7^ | 7.0±2.6 ×10^6^ |
|  | WT-1 | 3 | 3.6±1.5 ×10^3^ | 6.3±1.8 ×10^6^ | 5.5±2.8 ×10^6^ |
|  | **WT-2** | 3 | 1.0±0.7 ×10^4^ | 1.2±0.5 ×10^7^ | 4.9±1.3 ×10^6^ |
|  | **WT-3** | 3 | 5.3±4.0 ×10^5^ | 2.5±1.1 ×10^7^ | 2.0±1.2 ×10^7^ |

R, number of biological replicates
