# Supplementary material for: Experimental Virus Evolution Reveals a Role of Plant Microtubule Dynamics and TORTIFOLIA1/SPIRAL2 in RNA Trafficking
Source: PLoS One. 2014 Aug 18;9(8):e105364. doi: 10.1371/journal.pone.0105364 (PMC4136834; doi:10.1371/journal.pone.0105364)
Supplement: Table S2 — Accumulation of the TMV ancestor and evolved lineages in tobacco BY-2 protoplasts at 6, 12, 24, 48 and 72 hpi. (DOCX) [file pone.0105364.s003.docx]

Table S2. Accumulation of the TMV ancestor and evolved lineages in tobacco BY-2 protoplasts at 6, 12, 24, 48 and 72 hpi

|  |  | **Number of viral RNA copies (mean ± SE)** | | | | | |
| --- | --- | --- | --- | --- | --- | --- | --- |
| **Virus** | **R** | **6** | **12** | **24** | **48** | **72** | |
| Ancestor | 3 | 3.2±0.6 ×10^3^ | 6.4±2.4 ×10^4^ | 1.6±0.4×10^6^ | 7.1±0.9 ×10^6^ | | 1.8±0.2 ×10^7^ |
| **Tor1-1** | 3 | 4.5±1.7 ×10^3^ | 8.6±3.8 ×10^4^ | 2.1±0. 6×10^6^ | 6.6±1.4 ×10^6^ | | 1.2±0.2 ×10^7^ |
| **Tor1-2** | 3 | 1.0±0.5 ×10^4^ | 1.7±0.4 ×10^5^ | 3.5±1.4 ×10^6^ | 1.1±0.1 ×10^7^ | | 2.0±0.2 ×10^7^ |
| **Tor1-3** | 3 | 6.5±2.3 ×10^3^ | 6.7±0.7 ×10^4^ | 6.0±1.9 ×10^6^ | 7.4±2.4 ×10^6^ | | 1.5±0.1 ×10^7^ |
| **Tor2-1** | 3 | 1.3±0.5 ×10^4^ | 4.0±0.5 ×10^5^ | 4.6±1.2 ×10^6^ | 1.7±0.4 ×10^7^ | | 3.6±0.3 ×10^7^ |
| **Tor2-2** | 3 | 1.1±0.8 ×10^4^ | 1.3±0.3 ×10^5^ | 4.7±0.6 ×10^6^ | 13.3±0.7 ×10^6^ | | 5.2±1.1 ×10^7^ |
| **Tor2-3** | 3 | 4.3±0.5 ×10^3^ | 4.1±0.4 ×10^5^ | 3.5±0.9 ×10^6^ | 1.6±0.3 ×10^7^ | | 2.7±0.7 ×10^7^ |
| **WT-1** | 3 | 3.2±0.3 ×10^3^ | 9.6±1.0 ×10^4^ | 2.3±0.4 ×10^6^ | 7.8±1.0 ×10^6^ | | 1.5±0.3 ×10^7^ |
| **WT-2** | 3 | 6.6±2.9 ×10^3^ | 2.0±0.8 ×10^5^ | 2.7±04 ×10^6^ | 1.6±0.8 ×10^7^ | | 3.0±0.3 ×10^7^ |
| **WT-3** | 3 | 3.2±0.3 ×10^3^ | 6.9±0.2 ×10^4^ | 3.9±2.0 ×10^6^ | 1.2±0.3 ×10^7^ | | 2.0±0.4 ×10^7^ |

R, number of biological replicates.
