# Supplementary material for: Experimental Virus Evolution Reveals a Role of Plant Microtubule Dynamics and TORTIFOLIA1/SPIRAL2 in RNA Trafficking
Source: PLoS One. 2014 Aug 18;9(8):e105364. doi: 10.1371/journal.pone.0105364 (PMC4136834; doi:10.1371/journal.pone.0105364)
Supplement: Table S3 — Forward (F) and reverse (R) primers used for TMV genome amplification by RT-PCR and nucleotide sequencing. (DOCX) [file pone.0105364.s004.docx]

**Table S3.** Forward (F) and reverse (R) primers used for TMV genome amplification by RT-PCR and nucleotide sequencing.

| **Primer name** | **Sequence (5´-3´)** | **PCR product *** |
| --- | --- | --- |
| **TMV U1-1F** | GTATTTTTACAACAATTACCAAC | 1 - 1004 |
| **TMV U1-983R** | TCTAGAAAACTTACAAAACCAG |  |
| **TMV U1-735F** | CATACGTGCTATGCCGCTTTCC | 735 - 1742 |
| **TMV U1-1721R** | ATTGTACATCACTTCCGTTTCT |  |
| **TMV U1-1443F** | ACTAAGCTTGCCGTTCTAAAGG | 1443 - 2503 |
| **TMV U1-2482R** | AGAGTTCTGAGTTTCGCCATGT |  |
| **TMV U1-2218F** | TATCTGCTGCGGTGTCGAATCT | 2218 - 3216 |
| **TMV U1-3195R** | CATCAGAGTATGTCTCGCCTTG |  |
| **TMV U1-2893F** | GAGTTTCAGGATTCCCGTACCC | 2893 - 3929 |
| **TMV U1-3908R** | ATCTGCGAGCTGGCCTATTGTT |  |
| **TMV U1-3735F** | AACTTTAACGCACCCGAGTTGT | 3735 - 4716 |
| **TMV U1-4695R** | CCCAATCCTTGATGTGTTTAGC |  |
| **TMV U1-4319F** | AGTTTGGAAACAAGGGCATAGA | 4319 - 5285 |
| **TMV U1-5264R** | GTTATAGCATAATTGGGAACGA |  |
| **TMV U1-5097F** | ATACGTCTGTTTAGCCGGTTTG | 5097 - 6072 |
| **TMV U1-6051R** | CCACCGTTGCGTCGTCTACTCT |  |
| **TMV U1-5452F** | GGAGGGCCCATGGAACTTACAG | 5452 - 6395 |
| **TMV U1-6375R** | TGGGCCCCTACCGGGGGTAAC |  |

*numbers correspond to nucleotide positions in the TMV genome
